# Beyond Uncertainty Factors: Protecting the Tails of the Bell Curve

**DOI:** 10.1289/ehp.121-a26

**Published:** 2013-01-01

**Authors:** Charles W. Schmidt

**Affiliations:** **Charles W. Schmidt**, MS, an award-winning science writer from Portland, ME, has written for *Discover Magazine*, *Science*, and *Nature Medicine*.

Back in 1988, Bernard Weiss, now professor emeritus at the University of Rochester Medical Center, calculated that if the mean IQ of a hypothetical population of 100 million people fell by 5 points, then the number scoring below 70—a threshold for requiring remedial assistance—would swell from 6 million to 9.4 million.^1^ Graphed on what’s known as a normal distribution in statistics, Weiss’ analysis revealed how a shift in the population mean—in this case for IQ but conceivably for other physical features such as weight, cholesterol levels, and attention span—can be particularly harmful to certain segments of society.

Shifting means don’t occur spontaneously, however—they have a cause. A hypothetical drop in mean IQ, for instance, might result from widespread elevation in blood lead levels, and an increase in mean weight might result in part from widespread dietary changes or exposure to obesogens. And some individuals have predisposing risk factors that make them uniquely sensitive to the effects of these environmental stressors. For these vulnerable populations, a shift in the mean, as evidenced by Weiss’ calculation, could have disproportionate consequences.

How to identify and then protect vulnerable subgroups has been a long-standing challenge for environmental risk assessment. Now risk assessors are starting to leverage new data coming from genomics, molecular epidemiology, and other fields in an effort to set targeted exposure limits that protect defined groups of people.

The questions being asked are similar to discussions around personalized medicine, says Bill Farland, senior vice president for research at Colorado State University and former director of the National Center for Environmental Assessment in the U.S. Environmental Protection Agency (EPA) Office of Research and Development. “How far can we go towards protecting specific subgroups as opposed to relying on one-size-fits-all approaches?” he asks. “We need to bring more science into the discussion, and that’s what the field is confronting.”

## Understanding Uncertainty Factors

The specific factors that dictate how an individual will respond to an environmental exposure are called effect modifiers. A child’s response to lead, for instance, can be exacerbated by prenatal exposure to tobacco smoke, which also is neurotoxic, according to David Bellinger, a professor of environmental health at the Harvard School of Public Health. Bellinger says the two agents likely produce synergistic effects.

Scientists have long known that effect modifiers influence responses to pollutants in both negative and positive ways, as evidenced by the observation that more stimulating environments may ameliorate some of lead’s cognitive effects.^2^ Today, however, the specific nature of these effect modifiers is becoming increasingly clearer. “We’ve gone from knowing that effect modifiers must exist to the point that we now have estimates of how big their effects are in defined groups of people,” says Joel Schwartz, a professor of environmental epidemiology, also at Harvard School of Public Health.

Ways to better incorporate effect modifiers were the topic of a meeting hosted by the National Academy of Sciences in Washington, DC, on 18–19 April 2012.^3^ Farland, who chaired the meeting, says opinions ranged from those who argued for simply lowering exposure levels in the name of caution to those who argued that better understanding and use of effect modifiers could lead to more targeted protective strategies.

**Why Should You Care about a Shifting Mean?**

**Figure:**
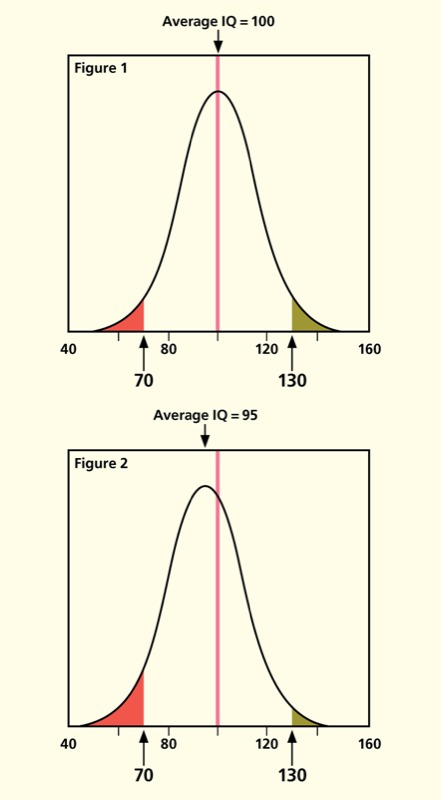
A 5-point loss in IQ might not affect an individual’s ability to live a productive life, but a 5-point shift in the population-level mean IQ could have profound implications for society. Why? An example published on the website www.ourstolenfuture.org and based in part on Weiss’ earlier work^1^ explains the scenario: “Imagine an unaffected population numbering 260 million people . . . with an average IQ of 100 and a standard deviation of 15 [Figure 1]. In that population there would be 6 million people with IQs above 130 and 6 million below 70. A decrease in average IQ of 5 points would shift the distribution to the left [Figure 2]. The number of people scoring above 130 would decline by 3.6 million while the number below 70 would increase by 3.4 million.”^^13^^ In other words, the number of people in the population categorized as “mentally retarded” would increase by 57%, and the number of people categorized as “gifted” would decrease by 60%. Figures adapted from Myers^^13^^

The standard way to protect sensitive subgroups has been to apply an “uncertainty factor” (UF) for interindividual variation toward the calculation of a health-based exposure limit. By applying such a UF—say, a factor of 10—a hypothetical reference dose of 100 mg/kg/day would drop to 10 mg/kg/day, thus increasing the margin of safety for anyone who might suffer adverse effects at higher exposure levels.

George Daston, a toxicologist and risk assessor at Procter & Gamble Company, in Cincinnati, Ohio, says regulators rely on a default 10-fold UF when they have limited information on subgroups that define the tails of the normal distribution. “Analyses of the literature support this as a protective approach, because in most cases the variability in response is less than what the default factor allows for,” he says.

“[UFs] allow us to make assumptions about the range of response variability,” Farland adds. “But in some cases they’re inadequate, and in others they’re overkill.”

According to Schwartz, the number and diversity of known effect modifiers continues to grow. Some are sociocultural. For instance, one study showed that resettled African refugees living in poor-quality housing in New Hampshire were unfamiliar with the concept of lead poisoning—which did not exist where they came from—making it difficult for them to understand the importance of protecting their children.^4^ Some are genetic, as occurs in the case of glutathione S-transferase gene variations that boost sensitivity to air pollutants.^5^ Some are medical—diabetics, for example, have a disproportionately high risk of heart attack when levels of particulate matter are high.^6^

In Bellinger’s view, effect modifiers for a given pollutant should be assessed independently so that their effects can be defined among people with, for instance, a predisposing genetic risk factor, or a co-occurring illness, or a psychosocial stressor. Then results from each of those studies can be incorporated into an interactive model that distinguishes among varying levels of risk.

“The assessment of effect modifiers should drive the study design,” Bellinger says. “As it stands now, analysis of potential effect modifiers is usually something tacked on at the end of the main study analyses to see if it is possible to account for unexplained variance in the association between the risk factor of primary interest and the health outcome.”

## Protecting Sensitive Subpopulations

Bellinger acknowledges that independent assessment of effect modifiers is a resource-intensive approach. But he says more simplistic alternatives might not accurately capture how exposures affect different groups of people. Schwartz agrees, pointing out that in his opinion, risk assessors need to identify the most susceptible people and then quantify their added level of risk so that policy makers can make appropriate management decisions.

These issues are highly relevant in the context of the EPA’s standard for ground-level ozone, Schwartz says, which has gotten snagged in disagreements between the agency, its Clean Air Scientific Advisory Committee (CASAC), and the White House. Citing growing evidence that ozone can be harmful to some people below the current standard of 75 ppb, the CASAC repeatedly urged the EPA to drop the standard to between 60–70 ppb averaged over 8 hours. As a basis for this recommendation, the CASAC stated that large segments of the population, namely children, the elderly, and people with chronic lung disease, are “intrinsically more susceptible” to ozone’s effects.^7^

In July 2011 the EPA forwarded a recommendation of 70 ppb to the Office of Management and Budget in its proposed final ozone rule,^8^ but on 2 September 2011, in response to widespread political opposition during an economic downturn (the change would have thrown many counties out of compliance with the Clean Air Act), President Barack Obama instructed the EPA to withdraw the more stringent standard.^9^ The EPA will reconsider the standard in 2013.

According to Schwartz, evidence shows that blacks, women, and people with asthma or atrial fibrillation have higher mortality risks at ozone levels significantly below the current standard.^10^ “The question is, are we willing to set standards that leave a small percentage of the population with a high risk of heart attack or death?” Schwartz asks. “We can demonstrate that the risk to these sensitive subgroups exists, but convincing people to spend billions to avoid it isn’t easy.”

To that, Weiss adds that more stringent environmental standards can generate huge economic benefits. He cites an EPA study showing that the benefits of phasing out leaded gasoline exceeded the costs 10 to 1, as measured by lifetime earnings from higher IQ combined with health savings from a commensurate drop in the incidence of cardiovascular disease.^11^

Still, Farland points out that from an economic perspective, it is impossible to protect all individuals. Policy makers can either try to “lop the tails off the distribution” with intervention strategies that target the most vulnerable people, he says, or they can “shift the curve” by lowering exposure standards or taking chemicals out of commercial circulation altogether.

“You want to be sure you’re protecting that ninety or ninety-nine percent of the population, and for the rest, it’s important that they understand what impacts their susceptibility,” Farland explains. “Asthmatics who jog, for instance, should be made aware of the potential risk if they exercise outside on days when the air quality is bad.”

Daston agrees that if risk mitigation strategies can be developed for specific sensitive subgroups, then it might not be necessary to lower exposure limits for the whole population. To illustrate, he cites the example of people born with phenylketonuria, who are unable to break down an amino acid, phenylalanine, that can cause adverse health effects if enough of it accumulates in the body. People with phenylketonuria can suffer toxic reactions to the artificial sweetener aspartame, which contains phenylalanine.^12^ “Individuals know they have the disorder, and so they avoid aspartame, but for the rest of the population, it’s safe,” he says. “But if there’s no way to limit exposure to a sensitive subgroup specifically, then exposure limits need to be set that are protective for that group.”

## Overhaul Needed?

But ultimately the insights and advances now coming out of research compel a restructuring of the whole risk assessment paradigm, says William Suk, director of the Center for Risk and Integrated Sciences at the National Institute of Environmental Health Sciences. Suk opines that the paradigm hasn’t been revised substantially for decades, and that the addition of more and more uncertainty factors (applied in certain cases to address children’s risk, or questions about interspecies variability in animal toxicology studies, or the difference between the lowest-observed-effect and no-effect levels, for instance) can eventually become unwieldy.

“How are we going to incorporate all these new risk factors?” he asks. “This is the challenge that we face now. And if it was easy, we would have figured out to do it already. It’s something we have to tackle in a more holistic way.”

Farland says regulatory strategies should include an educational component that emphasizes that regulating to zero risk is an impossible goal and that people can and should take actions to be sure they, or their families, are protected. He says, “Regulators make their best effort to protect the public within the constraints of law, but there also is an opportunity for the informed public to add to this protection.”
